# A detailed clinical and molecular survey of subjects with nonsyndromic *USH2A* retinopathy reveals an allelic hierarchy of disease-causing variants

**DOI:** 10.1038/ejhg.2014.283

**Published:** 2015-02-04

**Authors:** Eva Lenassi, Ajoy Vincent, Zheng Li, Zubin Saihan, Alison J Coffey, Heather B Steele-Stallard, Anthony T Moore, Karen P Steel, Linda M Luxon, Elise Héon, Maria Bitner-Glindzicz, Andrew R Webster

**Affiliations:** 1 UCL Institute of Ophthalmology and Moorfields Eye Hospital, University College of London, London, UK; 2 Eye Hospital, University Medical Centre, Ljubljana, Slovenia; 3 The Hospital for Sick Children, Department of Ophthalmology and Vision Sciences, University of Toronto, Toronto, ON, Canada; 4 Ocular Genetics, Singapore Eye Research Institute, Singapore, Singapore; 5 Wellcome Trust Sanger Institute, Hinxton, UK; 6 UCL Institute of Child Health, London, UK; 7 UCL Ear Institute, London, UK; 8 National Hospital for Neurology and Neurosurgery, London, UK

## Abstract

Defects in *USH2A* cause both isolated retinal disease and Usher syndrome (ie, retinal disease and deafness). To gain insights into isolated/nonsyndromic *USH2A* retinopathy, we screened *USH2A* in 186 probands with recessive retinal disease and no hearing complaint in childhood (discovery cohort) and in 84 probands with recessive retinal disease (replication cohort). Detailed phenotyping, including retinal imaging and audiological assessment, was performed in individuals with two likely disease-causing *USH2A* variants. Further genetic testing, including screening for a deep-intronic disease-causing variant and large deletions/duplications, was performed in those with one likely disease-causing change. Overall, 23 of 186 probands (discovery cohort) were found to harbour two likely disease-causing variants in *USH2A*. Some of these variants were predominantly associated with nonsyndromic retinal degeneration (‘retinal disease-specific'); these included the common c.2276 G>T, p.(Cys759Phe) mutation and five additional variants: c.2802 T>G, p.(Cys934Trp); c.10073 G>A, p.(Cys3358Tyr); c.11156 G>A, p.(Arg3719His); c.12295-3 T>A; and c.12575 G>A, p.(Arg4192His). An allelic hierarchy was observed in the discovery cohort and confirmed in the replication cohort. In nonsyndromic *USH2A* disease, retinopathy was consistent with retinitis pigmentosa and the audiological phenotype was variable. *USH2A* retinopathy is a common cause of nonsyndromic recessive retinal degeneration and has a different mutational spectrum to that observed in Usher syndrome. The following model is proposed: the presence of at least one ‘retinal disease-specific' *USH2A* allele in a patient with *USH2A*-related disease results in the preservation of normal hearing. Careful genotype–phenotype studies such as this will become increasingly important, especially now that high-throughput sequencing is widely used in the clinical setting.

## Introduction

Retinitis pigmentosa is the most common inherited retinal degeneration and a major cause of visual impairment among individuals aged 20–64 years.^1, 2^ It is genetically heterogeneous (over 60 genes implicated so far) and associated with significant variability in age of onset, disease progression and retinal appearance (RetNet; http://www.sph.uth.tmc.edu/retnet/, accessed 31 December 2014).^3^ Although retinitis pigmentosa is a disease confined to the eye, some 20–30% of patients have associated non-ocular disease; Usher syndrome, in which retinitis pigmentosa is combined with, typically prelingual, sensorineural hearing loss, is the most frequent syndromic form.^4^

Disease-causing variants in the *USH2A* gene are the most common cause of Usher syndrome (29% of all cases) and one of the most common causes of nonsyndromic autosomal recessive retinitis pigmentosa (19–23% of all cases).^4, 5^ The *USH2A* gene is located on 1q41 and has two alternatively spliced transcripts: a short one consisting of 21 exons, and a longer one consisting of 51 additional 3′ exons; the latter encodes a 5202 amino-acid matrix protein expressed specifically in photoreceptors and developing cochlear hair cells.^6, 7^ It has been shown that the USH2A protein is required for long-term maintenance of retinal photoreceptors and the development of cochlear cells.^7^

Over 2900 coding variants have been reported in the *USH2A* gene (1000 genomes project database, National Heart, Lung and Blood Institute Exome Sequencing Project or NHLBI ESP and LOVD-USHBase, accessed 15 September 2014). Over 470 of these changes are presumed to be pathogenic (HGMD, accessed 15 September 2014); most of these disease-causing variants are found in one or a few cases each, with the exception of c.2299delG, p.(Glu767Serfs*21) and c.2276 G>T, p.(Cys759Phe), which are more commonly found in patient cohorts. The c.2299delG variant causes a substantial proportion of cases of Usher syndrome,^8^ while the c.2276 G>T change has been associated mainly with disease confined to the eye.^9, 10^ This suggests the existence of alleles that are likely to be specific to those with nonsyndromic disease (‘retinal disease-specific'). However, this has not been studied in detail and to date no other such ‘retinal disease-specific' alleles have been identified.

Here, we have performed a comprehensive screen of the *USH2A* gene in 186 patients with autosomal recessive retinal degeneration and no complaint of childhood hearing loss to gain insights into nonsyndromic *USH2A*-related disease. Detailed phenotyping, including fundus autofluorescence imaging and audiological assessment, was performed in individuals found to harbour two likely disease-causing variants. The effect of three splice site changes on nasal mRNA was investigated and multiplex ligation-dependent probe amplification (MLPA) was performed in selected patients with the aim of detecting deletions and duplications in *USH2A*. Additionally, we have investigated whether nonsyndromic *versus* syndromic disease can be explained by the presence of an allelic hierarchy of *USH2A* disease-causing variants, and have addressed this by studying *USH2A* allelic heterogeneity in the discovery (*n*=186) and two additional (*n*=84 and *n*=187) cohorts.

## Materials and methods

### Study subjects

A total of 186 unrelated individuals with recessive retinal degeneration and no hearing complaint in childhood were ascertained from the clinics of Moorfields Eye Hospital (London, UK; discovery cohort). Of these, 168 patients were affected with rod–cone dystrophy (ie, retinitis pigmentosa), 12 with cone–rod dystrophy and 6 with childhood-onset retinal dystrophy. Further to this discovery cohort, 84 additional probands with recessive retinal degeneration (62 with nonsyndromic disease and 22 with Usher syndrome) were recruited at the Hospital for Sick Children (Toronto, ON, Canada); both cohorts underwent full sequencing of *USH2A* (see below). A third cohort of 187 unrelated patients with nonsyndromic, adult-onset, retinitis pigmentosa was also ascertained from the Moorfields Eye Hospital. This cohort was only used to test for selected variants in exons 13, 51, 57 and 63. A complete eye examination was performed and a detailed clinical history was obtained in all patients. Only patients with a family history compatible with autosomal recessive inheritance (ie, no evidence of dominant inheritance) were studied. For the purposes of this study, we define the phenotype observed in retinitis pigmentosa patients without prelingual/childhood-onset hearing loss as nonsyndromic disease. Subjects with Usher syndrome were not tested for common genetic causes of nonsyndromic hearing impairment.

After informed consent was obtained, blood samples were collected and genomic DNA was extracted from peripheral blood leucocytes. Control DNA and mRNA were obtained from consented unrelated healthy individuals. All investigations were conducted in accordance with the Declaration of Helsinki principles. Institutional Review Board (IRB)/Ethics Committee approval was obtained from the Moorfields Eye Hospital and the Hospital for Sick Children local ethics committees.

### *USH2A* screening and method used to distinguish disease-causing variants

The 186 probands with recessive retinal degeneration and no hearing complaint in childhood and the 84 probands with recessive retinal degeneration underwent bidirectional DNA sequencing of the 72 exons of the *USH2A* gene. Primers were designed for all exons and intron–exon boundaries of the transcript with accession number NM_206933.2 (a total of 105 primer pairs; genomic reference sequence NG_008212.3).^8^ The additional panel of 187 unrelated individuals with nonsyndromic, adult-onset, recessive retinitis pigmentosa underwent bidirectional DNA sequencing of exons 13, 51, 57 and 63 of the *USH2A* gene; these exons were the location of presumed ‘retinal disease-specific' variants. In 24 patients from the discovery cohort, only one likely disease-causing *USH2A* variant was identified. These patients were: (i) sequenced for the *USH2A* c.7595-2144 A>G, p.Lys2532Thrfs*56 change, which causes inclusion of a pseudoexon;^11^ and (ii) screened using MLPA to detect deletions and duplications in the *USH2A* gene. The SALSA MLPA FAM-labelled reagent kit with probe mixes P361-A1/P362-A2 developed by MRC-Holland (MRC-Holland, Amsterdam, The Netherlands) was used and reactions were performed according to the manufacturer's instructions. Two subjects with previously identified heterozygous deletions were included to act as positive controls and confirm the validity of the method.

Sequence alterations were classified as ‘likely disease-causing variants' if they (i) were either nonsynonymous (including missense) or coding insertions/deletions or splice site (positions ±3) or large duplications/deletions and (ii) have a minor allele frequency (MAF) of 0.15% or less in the NHLBI ESP data set (accessed 15 June 2014); this is the MAF of c.2276 G>T, the most common change identified in patients with recessive retinitis pigmentosa.^9^ The effect of synonymous variants on splicing was not assessed in the present study. All relevant data (variants and associated phenotypes) were submitted to the appropriate LOVD-USHBase, which can be accessed in http://www.LOVD.nl/USH2A (submission IDs: 0004401−0004452).

### Nasal epithelial mRNA analysis

Nasal epithelial mRNA analysis was performed in individuals carrying the variants c.12295-3 T>A (subject D13), c.9056-2 A>G (subject D10) or c.5776+1 G>A (subject D10). Nasal epithelial cell samples were obtained by gently brushing the lateral inferior turbinate with bronchial cytology brush (Diagmed Ltd, North Yorkshire, UK) and RNA was extracted from these samples using the NucleoSpin RNA II Extraction Kit (Macherey-Nagel, Duren, Germany) according to the manufacturer's guidelines. cDNA was reverse transcribed using a cDNA Synthesis Kit (BioLine, London, UK) with a random hexamer primer mix. For reverse transcriptase (RT)-PCR reactions, *USH2A* was amplified between exons 27 and 32, exons 45 and 49, exons 58 and 64 and exons 62 and 66. The housekeeping gene *β*-*actin* was amplified as a positive control. The identity of *USH2A* RT-PCR products was established by direct sequencing using standard procedures (primer sequences and conditions are available on request).

### Ophthalmological and audiological assessment

Detailed ophthalmological evaluation was performed in 23 probands with two likely disease-causing variants in *USH2A* (all from the discovery cohort); 4 affected siblings of the 23 probands were also assessed. Ophthalmological examination included best-corrected visual acuity testing, dilated fundus examination, colour fundus photography (TRC-50IA; Topcon, Tokyo, Japan), spectral domain optical coherence tomography (OCT) and fundus autofluorescence imaging. The Spectralis HRA+OCT with viewing module version 5.1.2.0 (Heidelberg Engineering, Heidelberg, Germany) was used to acquire tomographs in 24 patients; fundus autofluorescence images were acquired in 24 patients using the HRA2 and Spectralis HRA+OCT (over a 30° × 30° and/or a 55° × 55° field; Heidelberg Engineering) instruments.

Pure tone audiometry was conducted, in a sound-treated booth, using a calibrated GSI 61 audiometer with TDH 49 headphones to assess hearing thresholds^12^ in 19 patients with no complaint of hearing loss, who were found to have two disease-associated variants in *USH2A* (all from the discovery cohort); the method has been described previously.^8^ The audiology thresholds (0.25–8 kHz) were reviewed for right and left ear separately and compared with age- and gender-matched percentile bands of normative data.^13^ Patients were classified into three groups: Group 1 with normal hearing thresholds falling in the interquartile normative range for age and gender across all frequencies (1 A if all thresholds <40th percentile; 1B if thresholds fell in the 50–60th percentile band); Group 2 with high-frequency hearing thresholds (3–8 kHz) >75th percentile and markedly greater than low-frequency threshold (0.25–2 kHz) percentiles (ie, 30–70 percentile bands difference); and Group 3 with atypical/abnormal audiometric configurations^14^ and without other aetiological explanation.

## Results

### *USH2A* retinopathy is a major cause of adult-onset recessive retinal degeneration

In the discovery cohort (*n*=186), a total of 206 different sequence alterations were recorded in the exons and intron–exon boundaries of *USH2A*; 88 variants were missense, coding insertions/deletions or splice site changes. Of these, 52 were rare with an MAF <0.15% and thus were classified as likely disease-causing (21 were novel to this study and 31 were previously reported). Notably, 22 patients were found to harbour two of these likely disease-causing variants, whereas 24 were found to carry one likely disease-causing variant. In the latter group, one proband was found on MLPA testing to harbour a heterozygous duplication of exons 57–60. None of them was found to carry the c.7595-2144 A>G change, which causes inclusion of a pseudoexon.^11^

All 23 patients with two likely disease-causing variants were affected with adult-onset retinitis pigmentosa (Table 1). Therefore, 14% of patients with adult-onset recessive retinitis pigmentosa (23/168) were found to harbour two likely disease-causing variants in *USH2A*.

In the replication cohort of 84 patients with recessive retinal degeneration (syndromic and nonsyndromic), 25 additional probands with two likely disease-causing *USH2A* variants were identified. All 25 had a retinal phenotype consistent with retinitis pigmentosa; 11 of these had Usher syndrome type II and the remaining 14 reported no hearing complaint in childhood (Table 2).

When the third panel of 187 patients with nonsyndromic, adult-onset, recessive retinitis pigmentosa was sequenced for exons 13, 51, 57 and 63, the following variants were identified: c.2276 G>T (5 alleles); c.2299delG (3 alleles); c.2633 G>A, p.(Arg878His) (1 allele); c.10073 G>A, p.(Cys3358Tyr) (8 alleles); c.11156 G>A, p.(Arg3719Leu) (2 alleles); c.12575 G>A, p.(Arg4192His) (2 alleles); and c.12574C>T, p.(Arg4192Cys) (1 allele). One subject harboured the c.2276 G>T variant in homozygous state, a second subject had c.2276 G>T and c.12575 G>A, a third subject had biallelic c.2276 G>T and c.2299delG and two additional unrelated subjects carried the same pair of changes: c.2299delG and c.10073 G>A.

### Some *USH2A* alleles are only associated with nonsyndromic retinal disease

**Figure 1:**
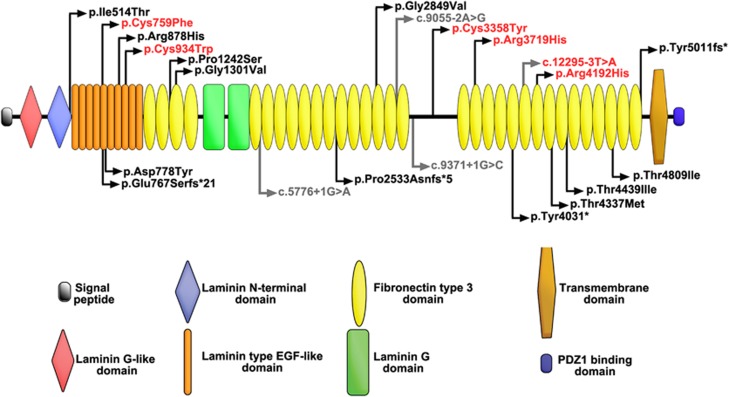
Schematic of the usherin protein and localisation of the likely disease-causing variants detected in the discovery cohort of patients (*n*=186); mutations previously reported in individuals with Usher syndrome type II are shown below the schematic. Presumed ‘retinal disease disease-specific' alleles are shown in red.

We define as ‘retinal disease-specific' variants or alleles that (i) were present in more than one patient with nonsyndromic retinal degeneration (in our discovery cohort and/or the literature) and (ii) have not been clearly associated with Usher syndrome type II to date (Table 3). On this basis, the following variants were categorised as likely ‘retinal disease-specific': c.2802 T>G, p.(Cys934Trp); c.10073 G>A; c.11156 G>A; c.12295-3 T>A; and c.12575 G>A. The c.2276 G>T variant that has been previously associated mainly with disease confined to the eye^9, 10^ was also included in this group. Notably, the most prevalent c.2276 G>T and c.10073 G>A variants were statistically significantly enriched in nonsyndromic cases compared with Usher syndrome type II cases (*P*=0.0060 and *P*=0.047, respectively (Fisher's exact test); the data on Usher syndrome type II were obtained from the UK National Collaborative Usher Study^8^). All ‘retinal disease-specific' variants were located in laminin-type EGF-like domains or fibronectin type 3 domains (Figure 1).

On examining our data, it was rare for nonsyndromic cases to have two ‘null' variants (ie, variants that are not missense and predicted to cause nonsense-mediated decay and/or significant truncation of the protein if translated), whereas this was common in those with Usher syndrome. Of 47 probands with nonsyndromic *USH2A*-related retinal degeneration (all three cohorts), only 5 had apparent biallelic ‘null' variants (this is the exception due to c.12295-3 T>C; see Table 1). This is significantly different to 39 out of 71 patients with *USH2A*-associated Usher syndrome^8^ (*P*=0.0001 (Fisher's exact test)). These data support the model that Usher syndrome represents the null phenotype consequent upon *USH2A* defects, and that ‘retinal disease-specific' alleles are partly functional, allowing them to contribute to normal cochlea development.

Assuming our model of allelic hierarchy is correct, further rare ‘retinal disease-specific' alleles can be sought. In those probands with nonsyndromic retinal disease, an allele is likely to be ‘retinal disease-specific' if either (i) it is homozygous or (ii) it is paired with an allele that has previously and consistently been reported to be associated with Usher syndrome or (iii) it is paired with an allele that has previously and consistently been reported to be associated with nonsyndromic retinitis pigmentosa. On examining our discovery and replication cohorts in this way, the following alleles are likely to be ‘retinal disease-specific': c.2332 G>T, p.(Asp778Tyr); c.3724C>T, p.(Pro1242Ser); c.4378 G>A, p.(Gly1460Arg); c.8546 G>T, p.(Gly2849Val); c.6904_6920dup17, p.(Gln2307Hisfs*25); c.12580 T>C, p.(Cys4194Arg) and c.15178 T>C, p.(Ser5060Pro). Further data from other cohorts of nonsyndromic patients are needed to confirm the ‘retinal disease-specific' nature of these alleles.

### The c.12295-3T>A, c.9056-2A>G and c. c.5776+1G>A variants result in abnormal *USH2A* pre-mRNA splicing

**Figure 2:**
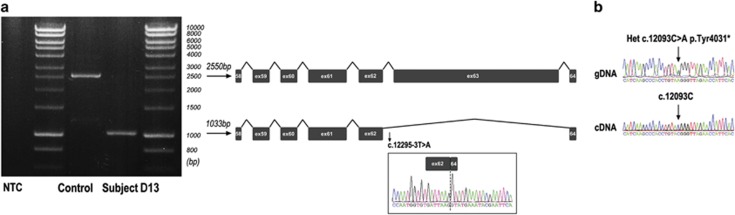
(**a**) RT-PCR analysis of the c.12295-3T>A mutation. RT-PCR was performed on RNA extracted from nasal epithelial cells of subject D13 and an unrelated control individual, using primers located in exons 58 and 64 of *USH2A*. In subject D13, RT-PCR produced a shorter product of 1033 bp corresponding to skipping of *USH2A* exon 63 (partial sequence chromatogram of this transcript is shown in the box; the dashed line indicates the splice junction between exons 62 and 64). The other allele of subject D13 harbouring a c.12093C>A, p.(Tyr4031*) mutation did not amplify. Amplification on control template produced a band of 2550 bp, corresponding to wild-type sequence. (**b**) Partial sequence chromatogram of genomic DNA from subject D13 showing a heterozygous c.12093C>A variant in exon 62. Sequence analysis of the corresponding RT-PCR product (1033 bp, see above) revealed a normal sequence at c.12093; this implies that the c.12295-3T>A and c.12093C>A variants reside on different alleles. NTC stands for no template control.

The effect of c.12295-3 T>A, one of the probably ‘retinal disease-specific' changes, on splicing was investigated in subject D13. Primers were used to amplify a 2550 bp fragment between exons 58 and 64. PCR products of the expected size were amplified for the control sample, whereas a much smaller band was observed in the patient sample. Further cDNA analysis (Figure 2a) revealed the presence of an abnormal transcript (1033 bp) associated with an out-of-frame skipping of exon 63; this would result in a premature termination codon. Amplification of a 2371 bp fragment between exons 62 and 66 combined with direct sequencing of the RT-PCR product confirmed the above findings. Notably, direct sequencing of the RT-PCR product demonstrated a normal sequence at position c.12093, where the sequencing of the genomic DNA identified a c.12093C>A, p.(Tyr4031*) change in heterozygous state. This suggests that the two likely disease-causing variants identified in this patient (c.12295-3 T>A and c.12093C>A) reside on two different alleles (Figure 2b). The non-amplification of the allele with c.12093C>A could be due to nonsense-mediated decay or preferential amplification of the smaller, exon-skipped PCR product from the other allele.

The effect of the c.9056-2 A>G (likely ‘retinal disease-specific') and c.5776+1 G>A (previously associated with Usher syndrome type II (LOVD-USHBase)) changes on splicing were investigated in subject D10; the c.9056-2 A>G variant led to part of exon 46 being missed in the mRNA, while the c.5776+1 G>A variant was associated with skipping of exon 28. These results are summarised in Supplementary Figure S1.

### Defects in *USH2A* consistently cause retinitis pigmentosa

The clinical features of 27 individuals with two likely disease-causing variants in *USH2A* (23 from discovery cohort plus 4 of their affected family members) are summarised in Table 1. All 27 patients were noted to have typical features of retinitis pigmentosa such as pigmentary changes in the midperipheral retina and vessel attenuation. Patients usually presented with nyctalopia (median age of 24.5 years; range 12–42 years). The median visual acuity at last visit was 0.24 logMAR (range −0.10 to 2.2). Six patients (22%) underwent cataract surgery at a median age of 47 (range 34–58) years. Central macular oedema was noted in 10 patients (37%) at a median age of 50 (range 35–59) years.

### Fundus autofluorescence imaging is a clinically useful test for *USH2A*-related disease

**Figure 3:**
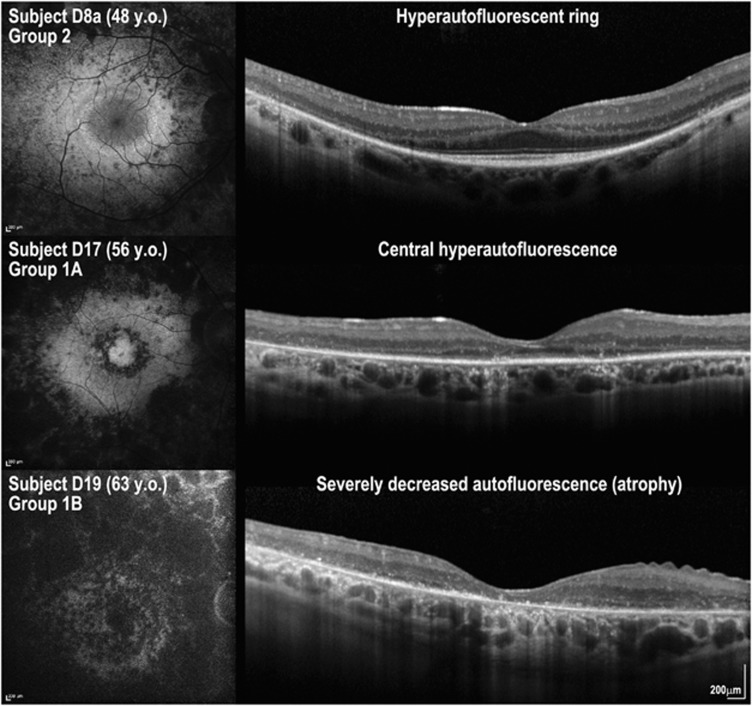
Fundus autofluorescence (FAF) imaging and foveal OCT scans of three patients with *USH2A* retinopathy. Three patterns were observed: (i) a hyperautofluorescent ring on FAF and preserved photoreceptor inner segment ellipsoid line in the area within the hyperautofluorescent ring on OCT (subject D8a; top row); (ii) central hyperautofluorescence on FAF and absent photoreceptor inner segment ellipsoid line on OCT (subject D17; middle row), and (iii) severely decreased autofluorescence on FAF and absent outer retina layers with thinning of the retinal pigment epithelium/Bruch's membrane complex band (subject D19; bottom row). Y.o., years old.

From the fundus autofluorescence images of 24 patients (48 eyes) three patterns were observed (Figure 3). Most patients (*n*=39 eye; 81.3%) showed preserved central autofluorescence surrounded by a variable diameter ring of high density (‘hyperautofluorescent ring' Figure 3, top row). Five (10.4%) eyes had an abnormally increased signal in the fovea with no obvious hyperautofluorescent ring (‘central hyperautofluorescence' Figure 3, middle row). Four (8.3%) eyes were characterised by widespread hypoautofluorescence corresponding to retinal pigment epithelial atrophy (‘severely decreased autofluorescence' Figure 3, bottom row). The findings were concordant between the eyes in all but two patients. Overlaying of fundus autofluorescence and OCT images suggested that the hyperautofluorescent ring represents a border between relatively preserved and diseased retinal tissue (Figure 3), and future structure–function correlation studies are expected to provide important insights into the clinical utility of this imaging modality

### Audiological phenotype in *USH2A* retinopathy is variable

**Figure 4:**
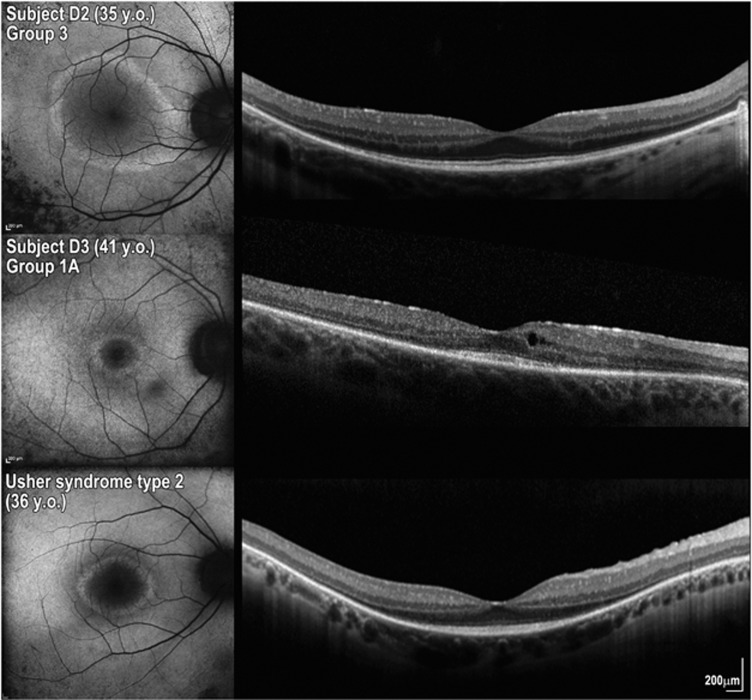
Variability in the severity of ocular and audiological phenotype due to mutations in *USH2A* in three patients of similar age. Fundus autofluorescence (FAF) imaging and foveal OCT show a better preserved retina in subject D2 (group 3 corresponding to abnormal audiological assessment) and in a patient with Usher syndrome type II compared with subject D3 (middle panel), who has normal hearing.

The 23 patients with two likely disease-causing variants from the discovery cohort and their four affected relatives reported no hearing loss in childhood, with 9 (33%) of these reporting subjective adult-onset hearing loss. Audiological assessment was conducted in 19 of these patients. In 14 (74%) patients, it was consistent with a Group 1 phenotype (ie, thresholds within normal limits); of those, 9 (47%) were classified in Group 1A and 5 (26%) in Group 1B. Three (16%) patients were classified in Group 2 and 2 (10%) in Group 3 (Supplementary Figure S2). Qualitative analysis revealed the c.12295-3T>A variant to be correlated with a more severe audiological phenotype (Groups 2 and 3). There seems to be no obvious correlation between the severity of visual and audiological phenotypes (Table 1, Figure 4 and Supplementary Figure S2).

## Discussion

**Figure 5:**
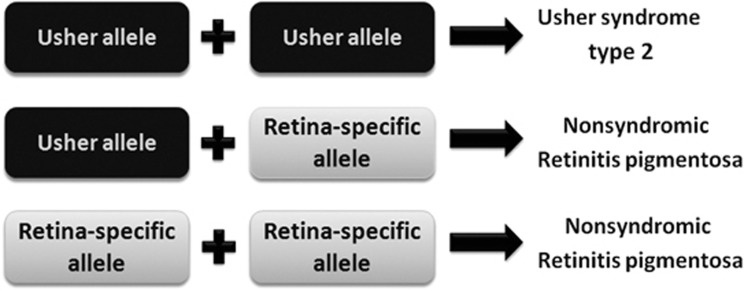
Schematic showing the proposed allelic hierarchy of *USH2A* mutations: the presence of at least one retinal disease-specific (‘retina-specific') *USH2A* allele in a patient with *USH2A*-related disease results in normal hearing at least in childhood.

In the present study, we confirm that recessive variants affecting USH2A function are a common cause of retinitis pigmentosa with disease-causing variants being spread throughout the gene. When allelic heterogeneity was studied and compared with that reported in Usher syndrome, the concept of ‘retinal disease-specific' *USH2A* alleles (ie, alleles associated with retinal degeneration and no hearing complaint in childhood) became apparent. The presence of at least one such allele in a patient with *USH2A*-related retinal degeneration results in relative preservation of hearing (Figure 5). Five likely ‘retinal disease-specific' variants (c.2802T>G; c.10073G>A; c.11156G>A; c.12295-3T>A and c.12575G>A) that are novel to this study were identified in addition to c.2276G>T, a relatively common sequence alteration previously associated with retinitis pigmentosa without hearing impairment.^9, 10^

Defects in the *USH2A* gene have been previously reported to account for 12–25% of all retinitis pigmentosa cases, dominant, recessive or X-linked; syndromic or nonsyndromic.^5^ McGee *et al*^5^ reported that among 80 patients with nonsyndromic recessive retinitis pigmentosa, 23% had one or two likely disease-causing variants in *USH2A*. This result was consistent with our findings: 24.7% (46/186) of patients with recessive retinal degeneration and no reported hearing loss in childhood harboured one or two likely disease-causing changes. Many variants in *USH2A* can be overlooked when only coding regions and intron–exon boundaries are sequenced. In previous studies, it was found that screening for duplications, deletions and a common deep-intronic sequence alteration (c.7595-2144A>G) detected a second disease-causing variant in 35% of cases with Usher syndrome type II that had only one variant affecting function on conventional Sanger sequencing of all *USH2A* exons.^11, 47^ In the present cohort, despite performing MLPA analysis and testing for the c.7595-2144A>G change, a significant number of cases with only one likely disease-causing in *USH2A* variant remained (23/186, 12.4%). This observation can be attributed to (i) a number of variants affecting function still being overlooked and/or (ii) some changes defined here as likely disease-causing being rare benign polymorphisms.

The first report of an *USH2A* change being associated with recessive retinitis pigmentosa without hearing impairment was by Rivolta *et al*^9^ in 2000; this change was a G to T transversion in exon 13 (c.2276G>T).^9^ Since then many studies have confirmed this finding (Table 1) and the c.2276G>T variant is often considered to be the most common disease-causing variant in patients with nonsyndromic retinitis pigmentosa.^9, 10, 24^ In the present study, c.2276G>T was found in 6.5% (12/186) of cases; in all study subjects, it was *in cis* with a previously reported polymorphism c.2256T>C, p.(His752His), suggesting a common ancestral haplotype. However, previous reports have shown that the c.2276G>T can be a recurrent sequence alteration.^17^ We have found five additional changes that are also associated with retinitis pigmentosa and no hearing complaint in childhood (presumed ‘retinal disease- specific' variants). These include four previously reported missense variants (c.10073G>A,^5, 8, 23, 26, 41^ c.2802T>G,^44^ c.11156G>A^5^ and c.12575G>A^5, 8, 23, 26, 41^) and a splice site change (c.12295-3T>A^8^). We have shown that the latter results in exclusion of exon 63 and an out-of-frame deletion. Three out of four patients harbouring this variant had no auditory complaint, despite the presence of an abnormal auditory phenotype, which would suggest a long-standing or very slowly progressive neurosensory hearing impairment (Table 1).

Analysis of allelic heterogeneity in *USH2A* in our discovery cohort (23 probands with presumed *USH2A*-related retinitis pigmentosa and no hearing complaint in childhood) revealed that all but one (patient D9) patient harboured at least one ‘retinal disease-specific' or novel (presumed ‘retinal disease-specific') *USH2A* allele (Table 1). Importantly, a similar pattern (Figure 5) was observed in our replication cohort (16 probands with presumed *USH2A*-related retinitis pigmentosa and no hearing complaint in childhood; Table 3). Only in one case (patient D9; Table 1) there appeared to be discordance: a c.2332G>T change was identified in homozygous state in a Somali patient with retinitis pigmentosa and normal audiometric testing; previously, this change has been reported in heterozygous state in a patient with Usher syndrome type II.^35^ One explanation for this could be that this is a rare polymorphism. Recently, a similar allelic hierarchy has been reported for change affecting *CDH23* function: a ‘nonsyndromic deafness' *CDH23* allele *in trans* configuration with a ‘syndromic/Usher syndrome type I' *CDH23* allele preserves vision and balance in deaf individuals.^48^

Audiometric findings were within normal limits for most patients with two likely disease-causing *USH2A* variants and no hearing complaint in childhood (14 of 19 tested; Table 1). Notably, the severity of the retinal phenotype did not obviously correlate with the severity of the hearing impairment (Figure 4); this is in keeping with previous reports.^24^ Interestingly, the eldest subject D23 in the present study reported adult-onset hearing loss and had a hearing defect consistent with Usher syndrome type II at age 75 years. This is in keeping with the notion that recessive variants in *USH2A* cause a spectrum of hearing defects that range from an early-onset phenotype consistent with Usher syndrome type II to completely normal hearing.

We have shown that an allelic hierarchy of variants affecting USH2A function is likely with ‘retinal disease-specific' alleles being phenotypically dominant to ‘Usher syndrome type II' alleles. Although this finding has implications for counselling, the fact that *USH2A* disease-causing variants are often private makes prediction of the fully evolved phenotype challenging. In any case, the audiological phenotype in *USH2A*-related disease is highly variable and a multidisciplinary approach is often relevant even to cases without hearing complaints in childhood.

## Figures and Tables

**Table 1:** Clinical characteristics and genotype of the patients with *USH2A*-related disease without early-onset hearing loss (discovery cohort)

Subject; family ID	*Age* *; gender	*VA (LogMAR)*		*Presenting symptom (age)*	*Hearing loss*		*USH2A sequencing results* b	*FAF*	*Comments*	*Ethnicity*
		*RE*	*LE*		*Subjective*	*Audiology*^a^				
D1; gc4627	34; M	0.00	0.18	NA	No	NA	c.[2276G>T(;)13010C>T], p.[(Cys759Phe)(;)(Thr4337Met)]	NA	—	European
D2; gc16390	35; M	0.00	−0.10	Nyctalopia (23 y.o.)	No	Group 3	c.[2299delG(;)12295-3T>A], p.[(Glu767Serfs *21)][?]	Ring BE	—	European
D3; gc15522	41; M	0.18	0.18	Nyctalopia (17 y.o.)	No	Group 1 A	c.[2276G>T(;)13316C>T], p.[(Cys759Phe)(;)(Thr4439Ile)]	Ring BE	—	European
D4; gc5134	42; F	2.20	0.48	Nyctalopia (20 y.o.)	No	Group 1A	c.[**3724C>T**(;)**3724C>T**], p.[(**Pro1242Ser)**(;)**(Pro1242Ser)**]	Atrophy BE	—	South Asian
D5; gc17134	42; F	0.24	0.10	Loss of peripheral vision (26 y.o.)	No	Group 1A	c.[2276G>T(;)2276G>T], p.[(Cys759Phe)(;)(Cys759Phe)]	Ring BE	CMO	European
D6; gc16520	42; F	0.30	0.18	Nyctalopia (27 y.o.)	Yes	NA	c.[2276G>T(;)12575G>A], p.[(Cys759Phe)(;)(Arg4192His)]	Ring BE	CMO	European
D7; gc17055	43; M	0.48	0.60	Nyctalopia (15 y.o.)	Yes	NA	c.[2276G>T(;)**15031delT**], p.[(Cys759Phe)(;)(**Tyr5011fs**)]	Ring BE	—	European
D8; gc16986	47; F	0.00	0.00	Nyctalopia (36 y.o.)	No	Group 1A	c.[2299delG(;)10073G>A] p.[(Glu767Serfs*21)(;)(Cys3358Tyr)]	Ring BE	—	European
D8a; gc16986	48; F	0.18	0.18	Nyctalopia (42 y.o.)	No	Group 2		Ring BE	—	European
D8b; gc16986	58; F	0.60	0.60	Nyctalopia (38 y.o.)	Yes	Group 1B		Ring BE	CMO, IOL LE	European
D9; gc16172	48; M	−0.10	0.00	Nyctalopia (30 y.o.)	No	Group 1B	c.[2332G>T(;)2332G>T], p.[(Asp778Tyr)][(Asp778Tyr)]	Ring BE	—	African
D14a; gc5204	50; M	0.48	0.60	Nyctalopia (18 y.o.)	No	NA	c.[2276G>T(;)14426C>T], p.[(Cys759Phe)(;)(Thr4809Ile)]	Ring BE	CMO	European
D14; gc5204	55; F	0.22	0.40	Dark adaptation (32 y.o.)	Yes	Group 1B		Ring BE	CMO	European
D10; gc16891	51; F	0.72	0.68	Nyctalopia (23 y.o.)	No	Group 1A	c.[5776+1G>A(;)**9056-2A>G**], p.[?][?]	Small ring BE	—	European
D11a; gc1985	50; M	0.20	0.22	Nyctalopia (13 y.o.)	No	Group 2	c.[2299delG(;)12295-3 T>A], p.[(Glu767Serfs*21)][?]	Small ring BE	CMO, IOL BE	European
D11; gc1985	52; F	0.28	0.42	Nyctalopia (17 y.o.)	No	Group 2		Small ring BE	IOL BE	European
D12; gc15971	52; M	0.22	0.08	Dark adaptation (42 y.o.)	No	Group 1A	c.[7595-3C>G(;)**8546G>T**], p.[Pro2533Asnfs*5(;)(**Gly2849Val**)]	Ring BE	CMO	European
D13; gc860	54; M	0.18	0.18	Nyctalopia (14 y.o.)	Yes	Group 1B	c.[**12093C>A**][12295-3T>A], p.[(Tyr4031*)][?]	Small ring BE	IOL BE	European
D15; gc4654	55; M	0.18	0.18	Nyctalopia (32 y.o.)	Yes	NA	c.[10073G>A(;)11156G>A], p.[(Cys3358Tyr)(;)(Arg3719His)]	Ring BE	—	European
D16; gc16801	55; M	0.18	0.00	Nyctalopia (30 y.o.)	No	NA	c.[**2633G>A**(;)3902G>T], p.[(**Arg878His**)(;)(Gly1301Val)]	NA	—	South Asian
D17; gc16524	56; F	0.78	0.78	Nyctalopia (35 y.o.)	No	Group 1A	c.[2276G>T(;)10073G>A], p.[(Cys759Phe)(;)(Cys3358Tyr)]	Central hyperAF BE	IOL BE	European
D18; gc5399	58; M	0.36	0.20	Nyctalopia (12 y.o.)	No	Group 1A	c.[2276G>T(;)2299delG], p.[(Cys759Phe)(;)(Glu767Serfs*21)]	Small ring BE	CMO	European
D19; gc5396	63; F	1.30	0.60	Nyctalopia (15 y.o.)	Yes	Group 1B	c.[2276G>T(;)2299delG], p.[(Cys759Phe)(;)(Glu767Serfs*21)]	Atrophy RE; Central hyperAF LE	CMO	European
D20; gc1802	66; F	0.50	1.30	Nyctalopia (28 y.o.)	No	NA	c.[14219C>A(;)**11048-?_11711+?dup**], p.[(Ala4740Asp)][?]	Central hyperAF BE	—	European
D21; gc2053	68; M	2.20	2.20	Nyctalopia (15 y.o.)	No	Group 1A	c.[2802T>G(;)12575G>A], p.[(Cys934Trp)(;)(Arg4192His)]	Atrophy BE	—	European
D22; gc4737	69; M	−0.10	0.20	Nyctalopia (29 y.o.)	Yes	NA	c.[2276G>T(;)13010C>T], p.[(Cys759Phe)(;)(Thr4337Met)]	Small ring BE	IOL BE	European
D23; gc945	77; F	2.20	0.48	Nyctalopia (13 y.o.)	Yes	Group 3	c.[**1541T>C**(;)9371+1G>C], p.[(**Ile514Thr**)][?]	Atrophy RE; Small ring LE	CMO	European
Median (range)	52 (34, 77)	0.24 (−0.10, 2.20)	0.2 (−0.10, 2.20)	24.5 (12, 42)						

Abbreviations: BE, both eyes; CMO, cystoid macular oedema; FAF, fundus autofluorescence imaging; hyperAF, hyperautofluorescence, IOL, intraocular lens implants; LE, left eye; NA, not applicable; RE, right eye; VA, visual acuity.

*Age at last examination.

a *Audiology data*: Group 1 good hearing across all frequencies (1A if <40th percentile; 1B if 50–60th percentile); Group 2 marked high-frequency hearing loss compared with low-frequency percentiles; Group 3 atypical/abnormal audiometric configuration without other aetiological explanation. Notably, in subject D2, the audiogram, although abnormal, it was not consistent with Usher syndrome type II. Conversely, subject D23, who reported adult-onset hearing loss, had an audiogram consistent with Usher syndrome type II at age 75 years (see Discussion).

b Variants that are novel to this study are presented in bold. Of these novel changes, only c.3724C>T is found in the ExAC (Exome Aggregation Consortium) browser (3/122810 alleles; accessed 31 December 2014).

b Subjects D8, D8a and D8b; subjects D11 and D11a; and subjects D14 and D14a are siblings. Numbering of *USH2A* variants has been assigned in accordance with NCBI Reference Sequence NM_206933.2.

**Table 2:** Genotype and clinical characteristics of patients with *USH2A*-related disease (replication cohort)

*Subject*	*USH2A sequencing results*		*Likely effect of allele*		*Diagnosis*
	*Variant 1*	*Variant 2*	*Variant 1*	*Variant 2*	
R1	**c.2276G>T, p.(Cys759Phe)**	**c.2276G>T, p.(Cys759Phe)**	Retina-specific	Retina-specific	Nonsyndromic retinitis pigmentosa
R2	**c.2276G>T, p.(Cys759Phe)**	c.1225T>C, p.(Trp409Arg)	Retina-specific	Unknown (novel)	Nonsyndromic retinitis pigmentosa
R3	**c.2276G>T, p.(Cys759Phe)**	c.9912dup, p.(Glu3305Argfs*41)	Retina-specific	Unknown	Nonsyndromic retinitis pigmentosa
R4	**c.2276G>T, p.(Cys759Phe)**	c.2299delG, p.(Glu767Serfs*21)	Retina-specific	Usher	Nonsyndromic retinitis pigmentosa
R5	c.99_100insT, (p.Arg34Serfs*41)	**c.2802T>G, p.(Cys934Trp)**	Unknown	Retina-specific	Nonsyndromic retinitis pigmentosa
R6	c.5776G>A, p.(Glu1926Lys)	**c.10073G>A, p.(Cys3358Tyr)**	Usher	Retina-specific	Nonsyndromic retinitis pigmentosa
R7	c.5776G>A, p.(Glu1926Lys)	**c.10073G>A, p.(Cys3358Tyr)**	Usher	Retina-specific	Nonsyndromic retinitis pigmentosa
R8	c.1256G>T, p.(Cys419Phe)	**c.11156G>A, p.(Arg3719His)**	Usher	Retina-specific	Nonsyndromic retinitis pigmentosa
R9	c.1256G>T, p.(Cys419Phe)	**c.11156G>A, p.(Arg3719His)**	Usher	Retina-specific	Nonsyndromic retinitis pigmentosa
R10	c.11864G>A, p.(Trp3955*)	c.12580T>C, p.(Cys4194Arg)	Usher	Unknown (novel)	Nonsyndromic retinitis pigmentosa
R11	c.8254G>A, p.(Gly2752Arg)	c.15178T>C, p.(Ser5060Pro)	Usher	Unknown (novel)	Nonsyndromic retinitis pigmentosa
R12	c.4378G>A, p.(Gly1460Arg)	c.9424G>T, p.(Gly3142*)	Unknown (novel)	Usher	Nonsyndromic retinitis pigmentosa
R13	c.6904_6920dup17	c.12877G>A, p.(Gly4293Ser)	Unknown (novel)	Unknown (novel)	Nonsyndromic retinitis pigmentosa
R14	c.9611A>G, p.(His3204Arg)	c.13768G>A, p.(Gly4590Ser)	Unknown (novel)	Unknown (novel)	Nonsyndromic retinitis pigmentosa
R15	c.1876C>T, p.(Arg626*)	c.1876C>T, p.(Arg626*)	Usher	Usher	Usher syndrome
R16	c.1256G>T, p.(Cys419Phe)	c.2299delG, p.(Glu767Serfs*21)	Usher	Usher	Usher syndrome
R17	c.2299delG, p.(Glu767Serfs*21)	c.14287G>A, p.(Gly4763Arg)	Usher	Usher	Usher syndrome
R18	c.2299delG, p.(Glu767Serfs*21)	c.14287G>A, p.(Gly4763Arg)	Usher	Usher	Usher syndrome
R19	c.2209C>T, p.(Arg737*)	c.2299delG, p.(Glu767Serfs*21)	Usher	Usher	Usher syndrome
R20	c.2299delG, p.(Glu767Serfs*21)	c.5168-2A>G	Usher	Unknown (novel)	Usher syndrome
R21	c.2299delG, p.(Glu767Serfs*21)	c.5858-1G>A	Usher	Unknown (novel)	Usher syndrome
R22	c.2299delG, p.(Glu767Serfs*21)	c.14180G>A, p.(Trp4727*)	Usher	Unknown (novel)	Usher syndrome
R23	c.1679delC, p.(Pro560Leufs*31)	c.11549-1G>A	Unknown	Unknown (novel)	Usher syndrome
R24	c.854T>C, p.(Ile285Thr)	c.10724G>A, p.(Cys3575Tyr)	Unknown (novel)	Usher	Usher syndrome
R25	c.2081G>C, p.(Cys694Ser)	c.10612C>T, p.(Arg3538*)	Unknown (novel)	Unknown (novel)	Usher syndrome

Retina-specific corresponds to likely ‘retinal disease-specific' alleles (shown in bold).

Usher corresponds to likely ‘Usher syndrome type II'-specific alleles.

Novel corresponds to alleles that are novel to this study. Of these novel changes, the following are found in the Exome Aggregation Consortium (ExAC) browser (accessed 31 December 2014): c.12580T>C (1/122018), c.15178T>C (4/122952 alleles), c.4378G>A (3/122140) and c.9611A>G (22/122680). Segregation analysis was performed in subjects R12, R20 and R22 and has confirmed that the reported variants are *in trans*.

Numbering of *USH2A* variants has been assigned in accordance with NCBI Reference Sequence NM_206933.2.

**Table 3:** Summary of phenotypes associated with the previously reported *USH2A* variants that were identified in the present series

*Change in USH2A*	*Number of previously reported cases*						*References*
	*Nonsyndromic retinitis pigmentosa*	*Usher type II*	*Atypical Usher*	*Usher. type I*	*Usher type III*	*Asymptomatic*	
c.2276G>T, p.(Cys759Phe)	96 (12 hom)	14	5	1^a^	—	1 (hom)	^8,15, 16, 17, 18, 19, 20, 21, 22, 23, 24, 25, 26^
c.2299delG, p.(Glu767Serfs*21)	58	327 (46 hom)	6 (3 hom)	—	1	—	^8, 9, 10, 11, 15, 16, 17, 19, 20, 21, 22, 24, 25, 27, 28, 29, 30, 31, 32, 33, 34, 35, 36, 37, 38, 39, 40, 41, 42, 43^
c.2332G>T p.(Asp778Tyr)	—	1	—	—	—	—	^35^
c.2802 T>G, p.(Cys934Trp)	1	—	—	—	—	—	^44^
c.3902G>T, p.(Gly1301Val)	—	—	—	1*	—	—	^40^
c.5776+1G>A	—	4	—	—	1	—	^21, 24, 25, 40^
7595-3C>G, p.Pro2533Asnfs*5	—	5	—	—	—	—	^8, 20, 45^
c.9371+1G>C	—	1	—	—	—	—	^8^
c.10073 G>A, p.(Cys3358Tyr)	5	—	1	—	—	—	^5, 8, 23, 26, 41^
c.11156 G>A, p.(Arg3719His)	1	—	—	—	—	—	^5^
c.12295-3 T>A	—	—	1^b^	—	—	—	^8^
c.12575 G>A, p.(Arg4192His)	4 (1 hom)	—	1^c^ (hom)	—	—	—	^5, 8, 23, 26^
c.13010C>T, p.(Thr4337Met)	—	2	—	—	—	—	^5, 35^
c.13316C>T, p.(Thr4439Ile)	—	5	—	—	—	—	^8, 21, 46^
c.14426C>T p.(Thr4809Ile)	—	3	—	—	—	—	^8, 36^

Numbering of *USH2A* variants has been assigned in accordance with NCBI Reference Sequence NM_206933.2. The complete list of references can be found in LOVD-USHBase.

a A single heterozygous variant in *MYO7A* was also reported in this patient; *a homozygous (hom) variant in *MYO7A* was also reported in this patient.

b After reviewing the clinical data, the patient was categorised as atypical due to adult onset of hearing loss (45 years old) and normal speech.

c After reviewing the clinical data, the patient was categorised as atypical due to very mild, progressive hearing loss.
