# Supplementary material for: A detailed clinical and molecular survey of subjects with nonsyndromic USH2A retinopathy reveals an allelic hierarchy of disease-causing variants
Source: Eur J Hum Genet. 2015 Feb 4;23(10):1318–27. doi: 10.1038/ejhg.2014.283 (PMC4592079; doi:10.1038/ejhg.2014.283)
Supplement: Supplementary Information [file ejhg2014283x1.doc]

**SUPPLEMENTAL MATERIAL**

**A detailed clinical and molecular survey of subjects with nonsyndromic *USH2A*-retinopathy reveals an allelic hierarchy of disease-causing variants**

Eva Lenassi,^1,2^ Ajoy Vincent,^3^ Zheng Li,^1,4^ Zubin Saihan,^1^ Alison J. Coffey,^5^ Heather B. Steele-Stallard,^6^ Anthony T. Moore,^1^ Karen P. Steel,^5^ Linda M. Luxon,^7,8^ Elise Héon,^3^ Maria Bitner-Glindzicz,^6^ Andrew R. Webster,^1 *^

^1^UCL Institute of Ophthalmology & Moorfields Eye Hospital, London, United Kingdom

^2^Eye Hospital, University Medical Centre, Ljubljana, Slovenia

^3^The Hospital for Sick Children, Department of Ophthalmology and Vision Sciences, University of Toronto, Toronto, Canada

^4^Ocular Genetics, Singapore Eye Research Institute, Singapore

^5^Wellcome Trust Sanger Institute, Hinxton, United Kingdom

^6^UCL Institute of Child Health, London, United Kingdom

^7^UCL Ear Institute, London, United Kingdom

^8^National Hospital for Neurology and Neurosurgery, London, United Kingdom

* Correspondence: Professor Andrew R. Webster,

UCL Institute of Ophthalmology, 11-43 Bath Street, London EC1V 9EL, United Kingdom.

Tel: +44 7566 2260; Fax: +44 20 7608 6830; Email: andrew.webster@ucl.ac.uk

**Figure S1.**

**
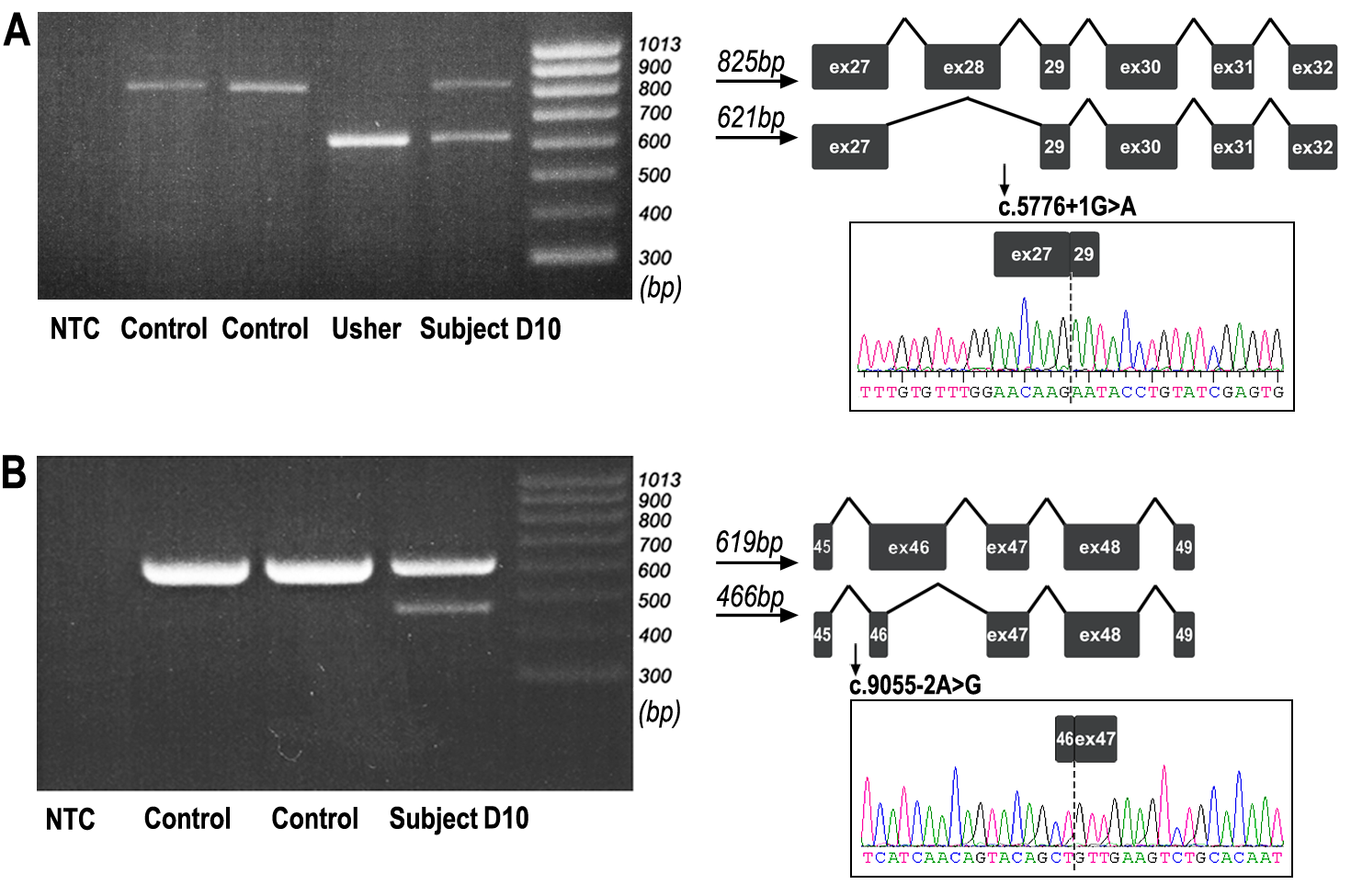
**

**A.** Reverse Transcription (RT) PCR analysis of the c.5776+1G>A variant in *USH2A*. RT-PCR was performed on RNA extracted from nasal epithelial cells of subject D10, an Usher syndrome patient (homozygous for c.5776+1G>A) and two unrelated control individuals, using primers located in exons 27 and 32 of *USH2A*. In subject D10, RT-PCR produced a shorter product of 621 bp corresponding to deletion of 204 bp *USH2A* exon 28 resulting in an in-frame deletion (partial sequence chromatogram of this transcript is shown in the box; the dashed line indicates the splice junction between exon 27 and 29) and a band of 825 bp corresponding to wild type sequence. In the Usher syndrome patient homozygous for c.5776+1G>A, only the shorter product of 621 bp was present. In controls, only the fragment of the expected size was present.

**B.** RT-PCR analysis of the c.9056-2A>G variant in *USH2A*. RT-PCR was performed on RNA extracted from nasal epithelial cells of subject D10 and two unrelated control individuals, using primers located in exons 45 and 49 of *USH2A*. In subject D10 RT-PCR produced a shorter product of 466 bp corresponding to deletion of the last 153 bp of *USH2A* exon 46 resulting in an in-frame deletion (partial sequence chromatogram of this transcript is shown in the box; the dashed line indicates the splice junction between exon 46 and 47) and a band of 619 bp corresponding to wild type sequence. Only the latter was present when amplifying control template. Interestingly, three splice site prediction tools (Human Splicing Finder (HSF), NNSPLICE and NetGene2) predict a splice donor site 153 nucleotides upstream of the genuine splice donor site in both the wild-type and mutant sequences.

**Figure S2.**

**
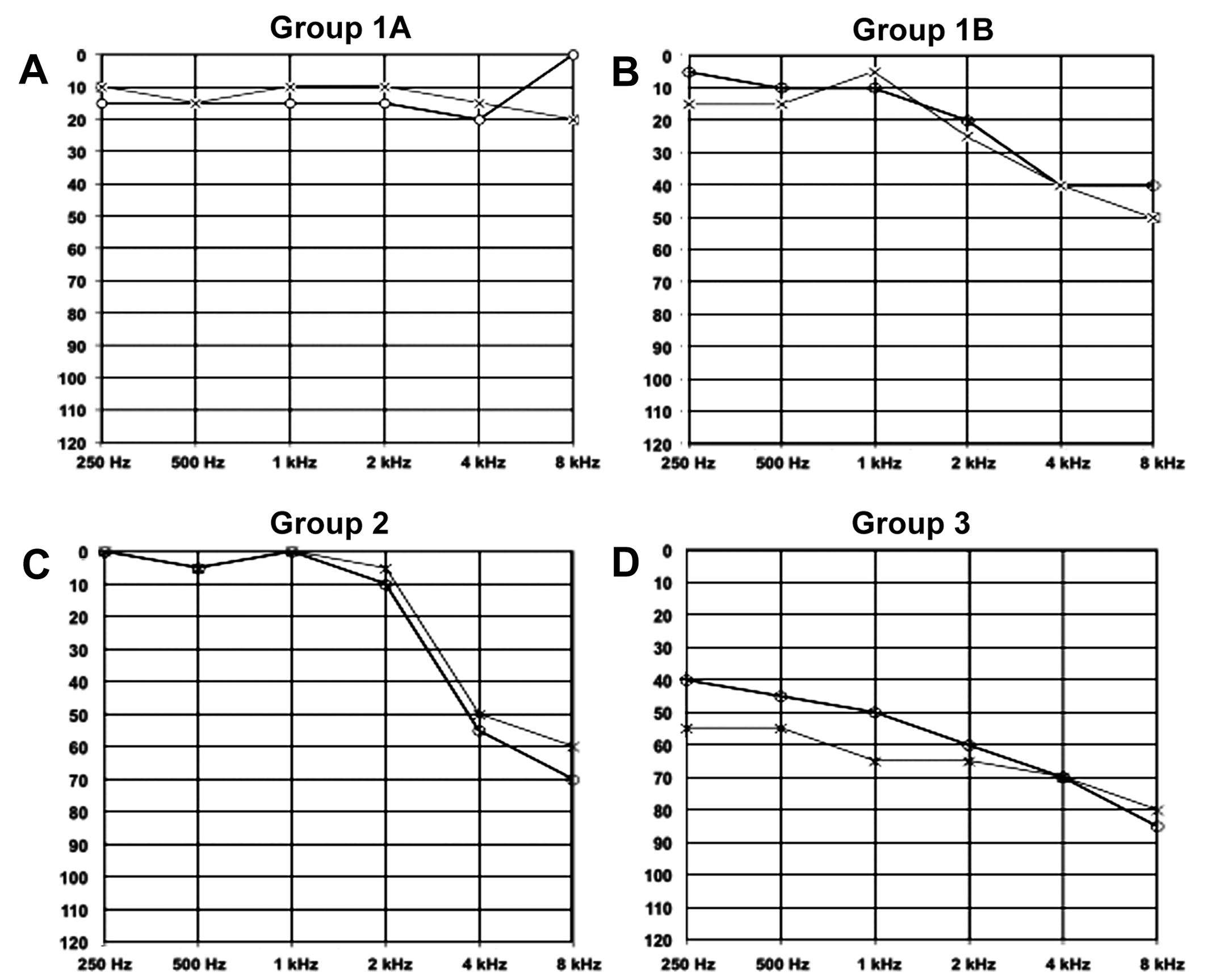
**

**A.** Typical pure tone audiogram of a patient (subject D17; female 56 years) in Group 1A with autosomal recessive retinitis pigmentosa, *USH2A* disease-causing variants and normal hearing with auditory thresholds of all frequencies ≤ 40th percentile for normal population.

**B.** Typical pure tone audiogram of a patient (subject D19; female 63 years) in Group 1B with autosomal recessive retinitis pigmentosa, *USH2A* disease-causing variants and normal hearing with auditory thresholds of all frequencies ≤ 60th percentile for normal population).

**C.** Typical pure tone audiogram of a patient (subject D11a; male 50 years) in Group 2 with autosomal recessive retinitis pigmentosa, *USH2A* disease-causing variants, high frequency hearing loss with auditory thresholds at high frequencies ˃75th percentile and a marked discrepancy at the low frequency and high frequency threshold percentile band compared with the normal population.

**D.** Pure tone audiogram of a patient (subject D23; female 77 years) in Group 3 with autosomal recessive retinitis pigmentosa, *USH2A* disease-causing variants, abnormal audiogram and hearing loss.
